# Development of a fully human glioblastoma-in-brain-spheroid model for accelerated translational research

**DOI:** 10.1016/j.jare.2025.03.055

**Published:** 2025-04-04

**Authors:** Sandra Horschitz, Ammar Jabali, Sophie Heuer, Eric Zillich, Lea Zillich, Dirk C. Hoffmann, Akshaya Senthil Kumar, David Hausmann, Daniel Dominguez Azorin, Ling Hai, Wolfgang Wick, Frank Winkler, Philipp Koch

**Affiliations:** a Central Institute of Mental Health, Heidelberg University/ Medical Faculty Mannheim, Mannheim, Germany; b Hector Institute for Translational Brain Research (HITBR gGmbH), Mannheim, Germany; c German Cancer Research Center, Heidelberg, Germany; d Department of Genetic Epidemiology in Psychiatry, Central Institute of Mental Health, Medical Faculty Mannheim, Heidelberg University; e Clinical Cooperation Unit Neurooncology, German Cancer Consortium (DKTK), German Cancer Research Center (DKFZ), Heidelberg, Germany; f Department of Neurology and Neurooncology Program, National Center for Tumor Diseases, Heidelberg University Hospital, Heidelberg, Germany; g Bioinformatics and Omics Data Analytics, German Cancer Research Center (DKFZ), Heidelberg, Germany

**Keywords:** Cortical spheroids, Glioblastoma, Neuro-oncology, Glioma heterogeneity, Cancer treatment

## Abstract

**Introduction:**

Glioblastoma (GBM) progression and therapeutic resistance are significantly influenced by complex interactions between tumor cells and the brain microenvironment, particularly neurons. However, studying these interactions in physiologically relevant conditions has remained challenging due to limitations in existing model systems.

**Objectives:**

Here, we present hGliCS (human glioma-cortical spheroid), a novel fully human brain tumor model that overcomes key limitations of current approaches by combining patient-derived GBM cells with mature human cortical neurons derived from induced pluripotent stem cells.

**Results:**

We demonstrate that GBM cells in hGliCS develop three critical hallmark features observed in patients: (i) formation of tumor microtubes enabling intercellular communication, (ii) establishment of neuron-glioma synapses, and (iii) development of an interconnected network with coordinated calcium signaling. Single-cell RNA sequencing reveals that tumor cells in hGliCS exhibit cellular heterogeneity and transcriptional profiles remarkably similar to those observed in mouse xenografts, including activation of key oncogenic pathways and neuronal-like features. Notably, while GBM cells showed substantial transcriptional adaptation to the neural environment, neurons maintained their core identity with only subtle alterations in glutamate signaling and structural gene expression. We validate hGliCS as a drug screening platform by demonstrating resistance patterns to standard chemotherapy and radiation similar to clinical observations. Furthermore, we show the model’s utility in testing standard and novel therapeutic compounds targeting cell proliferation and tumor-specific neurobiological features, respectively.

**Conclusion:**

This physiologically relevant human model system provides new opportunities for studying GBM biology and tumor-neuron interactions in a controlled environment. By bridging the gap between simplified *in vitro* systems and complex *in vivo* models, hGliCS represents a promising platform for therapeutic development and personalized medicine approaches in GBM treatment.

## Highlights

• First brain tumor model combining patient GBM cells with iPSC-derived cortical neurons.
• GBM cells form tumor microtubes and functional synapses with human cortical neurons.
• scRNAseq shows GBM heterogeneity matching profiles seen in mouse xenografts.
• Neurons maintain identity while GBM cells adapt to neural microenvironment.
• Model enables drug screening targeting tumor-neuron interactions and shows therapy resistance patterns.

## Graphical abstract

**Figure:**
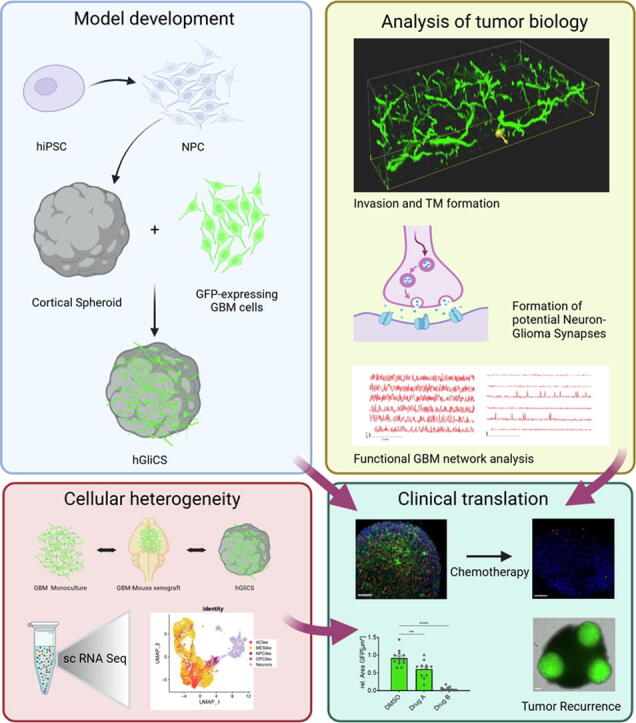

## Introduction

Glioblastoma (GBM) is an aggressive and highly malignant primary brain tumor that presents significant challenges in treatment and patient outcomes [1]. The complexity of this disease is rooted in its remarkable cellular heterogeneity and adaptive tumor microenvironment, which have consistently thwarted existing therapeutic approaches. Researchers have long struggled to develop experimental models that can comprehensively capture the intricate biology of GBM. Existing research models, such as 3D tumor organoids [2] and patient-derived xenografts [3,4], have demonstrated major limitations in addressing the multifaceted nature of the disease [[5], [6], [7]]. These models particularly fail to reconstruct the *in vivo*-like human tumor microenvironment [8]. The neuronal microenvironment actively shapes GBM progression through direct neuron-to-glioma synaptic connections and activity-dependent signaling [9,10]. GBM cells rapidly integrate into widespread neural circuits, with tumor-connected neurons actively promoting invasion and contributing to therapeutic resistance [11]. Understanding these interactions in physiologically relevant models is therefore essential for developing targeted therapies. Hence, the field has recognized the need for more sophisticated experimental systems that can more accurately represent the complex interactions within brain tumors [[12], [13], [14]].

In recent years, the development of 3D brain organoid models has emerged as a promising approach to study GBM cell interactions [[15], [16], [17]]. Cerebral brain organoids have shown particular potential in generating brain region-specific cell types and reflecting the cyto-architectural aspects of developing human brain tissue [18,19]. However, traditional brain organoids continue to present significant challenges, including the presence of substantial progenitor cell populations and the requirement for extended cultivation periods.

The critical nature of neuron-glioma interactions in understanding GBM is well established in the scientific community. These interactions are fundamentally dependent on neuronal activity, which necessitates experimental systems that can efficiently simulate mature neural environments. Existing models typically require extensive cultivation times to develop functional neuronal networks, creating a substantial barrier to comprehensive research and timely insights [20].

Our research introduces a novel experimental approach through the development of a human glioma-cortical spheroid (hGliCS) model. Unlike existing complex and heterogeneously structured organoids, our approach emphasizes standardized spheroids with synchronized differentiation and enhanced maturation through targeted small molecule interventions. By strategically departing from neurodevelopmental models, this approach offers a sophisticated platform for investigating glioma pathology in a more refined, physiologically relevant neural research system.

The hGliCS model offers unprecedented capabilities in particular for exploring GBḾs complex neuro-biological characteristics. It enables detailed investigation of critical GBM features, including diffuse invasion mechanisms [21], tumor microtube (TM) formation [22,23], autonomous cellular rhythmic activities [24], and the intricate interactions between neurons and glioma cells [9,10,25]. Moreover, the model successfully generates the tumor cell heterogeneity that is characteristic of GBM in clinical settings.

This approach represents a significant advancement in our ability to study and potentially treat one of the most challenging neurological cancers. By bridging critical gaps in current experimental methodologies, our hGliCS model provides a powerful new platform for investigating GBM, with potential implications for developing more targeted and effective therapeutic interventions.

## Materials and Methods

### Cell culture

Human induced pluripotent stem cells (hiPSCs) were reprogrammed from skin fibroblasts obtained from two healthy female probands with given informed consent (Ethics Committee II of Heidelberg University; approval no. 2009–350 N-MA). One hiPSC line is registered at www.hPSCreg.eu (CIMHi001-A). Both cell lines have been SNP genotyped to check for normal karyotypes and mycoplasma testing was performed on a regular basis.

Patient-derived primary GBM cell lines (S24, P3, BG5, T269) were obtained from adult patients diagnosed with glioblastoma after informed consent. Cultures of glioblastoma-initiating cells were cultured according to Lemke et al [26]. Briefly, tissue was washed, minced, and digested with trypsin, and then 100–500 mm pieces of tumor or brain ventricular tissue were washed in chilled sterile PBS (pH 7.4) with 0.6 % glucose. The tissues were minced with scissors and incubated in 0.1 % trypsin and 0.04 % DNase (Sigma type II) in Hanks balanced salt solution (HBSS) for 45 min at 37 °C. Tissues were washed three times in 0.04 % DNase in HBSS and then triturated in the same solution using first a 5-ml pipette then with a 1,000 Pipetteman (Rainin), and then a fire-polished Pasteur pipette. Cells were passed first through a 100 µm-strainer and then through a 70 µm-strainer. Cells were seeded at a concentration of 100,000 per ml into tissue culture flasks and cultivated in DMEM-F12 medium (Invitrogen, Cat#11330–032) under serum-free, non-adherent, ‘stem-like’ conditions [27] which includes 20 µl/ml B27 supplement (Invitrogen, Cat#12587–010), 5 μg/ml insulin (Sigma-Aldrich, Cat# I9278), 5 μg/ml heparin (Sigma-Aldrich, Cat# H4784), 20 μg/ml epidermal growth factor (rhEGF; R&D Systems, Cat#236-EG), and 20 μg/ml basic fibroblast growth factor (bFGF; Thermo Fisher Scientific, Cat# PHG0021). GBM cells generate brain tumors in mice that reflect different molecular disease subtypes and reflect the growth patterns of human incurable high-grade gliomas particularly well [9,22]. All cells were regularly tested for mycoplasma infections and species controls were performed for authenticity.

For cytosolic GFP expression, GBM cells were stably transduced with the pLKO.1-puro-CMV-TurboGFP_shnon-target-vector (custom made, Sigma Aldrich).

### Generation of human cortical spheroids

Neural induction: HiPSCs were cultured in Essential 8 (E8) medium on Geltrex^TM^ (Thermo Fisher Scientific) coated 6-well plates with daily medium changes. For the initial neural induction phase neural precursor cells (NPCs) were derived from hiPSCs by dual inhibition of SMAD signaling. The base medium consisted of advanced DMEM/F12 (Thermo Fisher Scientific, Cat#12634010), supplemented with B27 (1 %; Thermo Fisher Scientific, Cat#17504044), GlutaMax (1 %; Thermo Fisher Scientific, Cat#35050038), Pen/Strep (1 %; Thermo Fisher Scientific, Cat#15140122). This medium was further enhanced with the ALK2/3 inhibitor LDN-193189 (1 µM; StemCell Technologies, Cat#72148), the tankyrase inhibitor XAV (2 µM; Cell Guidance Systems, Cat#SM38-50) and the ALK4/5/7 inhibitor SB-431542 (10 µM; Cell Guidance Systems, Cat#SM33). Differentiation to NPC was started when hiPSC reached 70 % confluency by changing the medium to the neural induction formulation. After 4 days, cells were split 1:2 onto Geltrex coated 6-well plates in the same medium. Following another 4 days of culture, cells were split again at a 1:2 ratio, while the concentration of LDN-193189 was reduced to 200 nM and SB-431542 was omitted from the medium. A third 1:2 split was performed after an additional 5 days, and the medium was supplemented with FGF2 (20 ng/ml; Cell Guidance Systems, Cat#GFH28) while both, LDN-193189 and XAV were removed. Sphere formation: When the NPC reached full confluence 3–6 days later, they were dissociated into single cells using TrypLE Express and plated in a U-bottom shaped 96well-plate precoated with 0.5 % Pluronic. Each well received 100,000 cells in the same medium supplemented with 50 µM ROCK inhibitor Y-27632 (Cell Guidance Systems, Cat#SM02). This day was designated as day 0 of cortical spheroid culturing. Two days after plating, the cells had formed round-shaped neurospheres. Growth phase: These neurospheres were transferred into 6 cm dishes precoated with 0.5 % Pluronic. The medium was changed to DMEM/F12, 1 % B27, 1 % GlutaMax, 1 % P/S, and non-essential amino acids (1 %; Thermo Fisher Scientific, Cat#11140035). This medium was supplemented with the growth factors EGF and FGF2 (both 20 ng/ml; Cell Guidance Systems, Cat#GFH26 and GFH28) and the neurotrophin receptor co-activators LM22A (1 μM; Sigma-Aldrich, Cat#SML0848-25MG), and LM22B (1 μM; Tocris Bioscience, Cat#6037). The cortical spheroids were cultured in this medium for 18 days with medium changes every other day. The cultures were maintained under continuous agitation at 60 rpm on an orbital shaker (Infors Celltron HD) in an incubator set at 37 °C and 5 % CO_2_. Maturation phase: After the 18-day expansion phase, the growth factors EGF and FGF2 were omitted from the medium. Neuronal maturation of the cortical spheroids was promoted by supplementing the medium with 200 μM ascorbic acid, 1 μM LM22A, 1 μM LM22B, 2 μM of the CDK inhibitor PD-0332991 (Selleck Chemicals, Cat#1116), 5 μM of the γ-secretase inhibitor DAPT (Cell Guidance Systems, Cat#SM15-50), 3 μM of the GSK3 inhibitor CHIR99021 (Cell Guidance Systems, Cat#SM13-50), 10 μM of the adenylyl cyclase activator Forskolin (Cell Guidance Systems, Cat#SM18-100), and 300 μM GABA (Sigma-Aldrich, Cat#A5835-10G). The medium was changed twice per week to maintain optimal conditions for neuronal development. After 4 days of this maturation phase, the base medium was changed to Neurobasal containing 1 % B27, 1 % Pen/Strep, 1x GlutaMAX, and 5 mg/ml D-glucose to better promote neuronal survival. During the next scheduled medium change, DAPT, forskolin, and GABA were withdrawn from the formulation. One week later, CHIR99021 was also removed from the medium, completing the step-wise maturation protocol for the human cortical spheroids.

### Generation of hGliCS model

At least seven days prior to GBM coculture start medium of cortical spheroids was changed to advanced MEM (Thermo Fisher Scientific, Cat#12492013), B27 (1:100), GlutaMax (1 %), D-glucose (5 mg/ml), Pen/Strep (1 %) without LM22A, LM22B, PD-0332991, and ascorbic acid to improve neuronal functionality. GBM cells were dissociated into single cells using accutase and 5000 GBM cells were plated on top of one cortical spheroid per well of a U-bottom shaped 96 well-plate precoated with 0.5 % Pluronic in the same medium supplemented with EGF and FGF2 (20 ng/ml, each). The day of GBM cell addition was counted as d0 of hGliCS culture. After two days, cocultures were transferred to a 6 cm cell culture dish precoated with 0.5 % Pluronic. Medium was changed every other day. After 7 days of coculture, the growth factors EGF and FGF2 were withdrawn and GBM-cell-infiltrated cortical spheroids (hGliCS) were further cultured in this medium until the end of each experiment.

### Fixation, embedding, cryosectioning, immunocytochemistry

Processing of the cortical spheroids or hGliCS was performed as previously described [28]. In brief, spheroids were fixed in 4 % PFA for 20  min at room temperature and allowed to sink in 30 % sucrose at 4 °C overnight before being embedded in 10 %/7.5 % gelatine/sucrose in PBS and cryosectioned at 20 μm (Epredia, CryoStar NX50). For immunostaining of cryosections, samples were transferred to blocking solution (10 % horse serum in PBS) containing 0.5 % Triton X-100 for 1 h. Samples were then incubated with primary antibodies in blocking solution overnight at 4 °C, washed three times, incubated with secondary antibody for 45 min, counterstained with DAPI and mounted with Mowiol 4–88. Supplementary Table S2 provides an overview of all antibodies used in this study.

### Imaging and image quantifications

*In vitro* imaging: *In vitro* images were acquired on a CellDiscoverer 7 (Zeiss) and processed with the ZEN 3.1 (blue edition) software.

Epifluorescent microscopy: Images of stained samples were taken on a Leica DM6B fluorescent microscope with a plan-apochromat 10x/0.32 or 20x/0.8 objective with the same intensities for same filter cubes.

3D-reconstructions: For the quantification of nuclear marker proteins, z-stacks were converted to an Imaris file format and analysed with Imaris (v.9.9.1). Images were automatically reconstructed into a multi-channel 3D model. In a first step, DAPI-positive nuclei were detected in the blue channel with the Spot detection tool. Threshold settings for filter quality and region threshold were kept identical for all images of the same experiment. In a second step, nuclear marker signals were detected with the Spot detection tool in their respective channel with same threshold settings within one experimental outline.

For the quantification of GBM cells, the Surface detection tool of Imaris was used. GFP staining was analyzed in the green channel with following settings: Surface grain size: 0.656 µm, diameter of largest sphere: 15 µm, number of voxels Img = 1: 10. The manual threshold was kept identical for all images within the same experiment.

Confocal microscopy: Confocal images in Fig. 2D were acquired using a Zeiss LSM 710 ConfoCor 3 confocal microscope, controlled by ZEN Software (black edition, 2012). To accommodate different resolution requirements, two objectives were employed: initially, a 20x/0.8 plan-apochromat dry objective was used for broader area scanning and preliminary localization of regions of interest. Subsequently, detailed imaging was conducted with a 63x/1.40 oil DIC M27 plan-apochromat objective to achieve high-resolution images of the targeted regions.

Confocal images in Fig. 2E were acquired using a Leica Stellaris 5 equipped with a supercontinuum wide line laser and with software LAS X 4.7.0.28176. Z-stacks were taken with a plan-apochromat 63x/1.40 oil objective using different detectors for channels 555 and 568 both with the application of TAU gating (spectral separation based on photon arrival time).

### Clearing

Cortical spheroids or hGliCS were cleared with benzyl alcohol and benzyl benzoate (BABB) according to Renner et al [29]. Briefly, spheroids were transferred to 1.5 ml-tubes and fixed in 4 % PFA for 15 min at room temperature, washed with PBS and permeabilized for 1 h at 37 °C in 0.5 % Triton X-100 in PBS. Staining with primary antibodies was performed in blocking solution (6 % BSA in PBS supplemented with 0.5 % Triton X-100, and 0.1 % NaN_3_) for 6 days at 37 °C with gentle shaking. Antibody solution was exchanged every second day. On day 6 samples were washed in 0.1 % Triton X-100 in PBS 5 times for every 1 h. Secondary antibodies were mixed in blocking solution and stained for 6 days at 37 °C with gentle shaking and exchanging the solution every second day. Followed by washing as mentioned above in 0.1 % Triton X-100 in PBS. In order to dehydrate the samples, they were incubated in 15 min-steps at room temperature in PBS containing 25 %, 50 %, 75 %, 90 %, 100 % methanol, respectively. After 100 % methanol, samples were transferred to a BABB-resistant plate (Screenstar, Greiner, Cat# 655866) followed by incubation with a 1:1 (v/v) mixture of BABB/methanol for 30 min. Finally, adding a 1:1 (v/v) mixture of BABB. Samples were imaged on an Opera Phenix spinning disc microscope (Revvity) with a 40x/1.15 water objective and analyzed with Harmony 5.1 software.

### Calcium imaging

For calcium imaging experiments of neurons in cortical spheroids, spheroids were generated from the hiPSC line CIMHi001-A transduced with the genetic Ca^2+^ sensor pAAVS1-PC-GCaMP6f (addgene #73503). At least one week before the experiment medium was changed to advanced MEM, B27 (1:100), GlutaMax (1 %), D-glucose (5 mg/ml), Pen/Strep (1 %). Cortical spheroids were sliced on a vibratome (Leica VT1000S) to 150 µm-slices one day before the experiment. Slices were incubated in 24well plates at 37 °C and 5 % CO_2_. Recordings were performed in the same plate first in imaging buffer (25 mM HEPES pH 7.4, 10 mM Glucose) and after two washings and 20–30 min incubation in prewarmed stimulation buffer (25 mM HEPES pH 7.4, 150 mM NaCl, 8 mM KCl, 1 mM MgCl_2_, 10 mM Glucose, 4 mM CaCl_2_; based on Sun&Südhof [30]. Imaging was carried out at 37 °C and 5 % CO_2_ in a heat-controlled chamber using a Zeiss CellDiscoverer 7 with a 20x/0.7 dry objective at a 0.5x digital zoom. Time-lapse image sequences were acquired at 170-ms intervals for 3 min with an average frame of 870 per position. For each batch, all images were acquired using the same light intensity and exposure time. At least 2 positions per slice were recorded. At the end of the experiment 1 µM Tetrodotoxin (TTX) was added in order to block voltage-gated sodium channels and action potential generation with an incubation of 30 min and recordings of at least 4 positions in total were performed. Average fluorescence intensity of 70–100 manually selected region of interest (ROIs) per position for every timepoint were calculated with the ZEN 3.1 (blue edition) software. Several different variables for calcium signaling events were determined using the MATLAB script PeakCaller [31] (required rise/fall = 10 %, max lookback/lookahead = 25 pts measured as absolute percentage; trend control: finite difference diffusion [[2], [3], [4], [5], [6], [7], [8], [9], [10], [11], [12], [13], [14], [15], [16], [17], [18], [19], [20], [21], [22], [23], [24], [25], [26], [27], [28], [29], [30], [31], [32], [33], [34], [35], [36], [37], [38], [39], [40], [41], [42], [43], [44], [45], [46], [47], [48], [49], [50], [51], [52], [53], [54], [55], [56], [57], [58], [59], [60], [61], [62], [63], [64], [65]], trend smoothness = 10), including the total number, the mean number, the mean height, and the mean amplitude of signaling events.

For calcium imaging of GBM cells in hGliCS, S24 cells were transduced with the pHAGE-RSV-tdTomato-2A-GCaMP6s vector (#80316, addgene). Recordings of unsliced hGliCS were performed in flat-bottom 96well plates (Screenstar; Greiner) in prewarmed stimulation buffer [30] after 20–30 min pre-incubation in a humidity-controlled Opera spinning disc microscope (Revvity) at 37 °C and 5 % CO_2_ with a 20xWater objective (NA 1.0), excitation at 488 nm for 100 msec and a binning of 3. Time series were acquired for 1000 time points with a rate of 1.52 frames per second. Subsequently, 100 µM of the gap junction inhibitor MFA (BioMol Cayman Chemicals, Cay70550-5000) was added in stimulation buffer and hGliCS were incubated for 30 min at 37 °C before recording. The recordings were analyzed with Harmony 5.1 software. Here, the software automatically finds the cell region based on the fluorescence signal in the 488 nm channel. Within each cell region the fluorescence intensity per mean intensity (ΔF/F) was calculated per timepoint, exported and plotted with GraphPad 10.1.2.

### Single cell RNA sequencing (scRNAseq)

hGliCS or cortical spheroids of the same age (without GBM cells) were manually cut into pieces 28 days after starting the hGliCS culture and further dissociated for 15 min at 37 °C in papain solution (Sigma Aldrich) containing papain buffer (5 mM L-cysteine and 0.5 mM EDTA in Earlés balanced salt solution), 20 units papain (Sigma Aldrich, Cat#P3125), and 10 µg/ml DNase I (Sigma Aldrich, Cat#10104159001). Excess papain solution was aspirated, replaced by 2 ml prewarmed coculture medium and cells were mechanically dissociated by pipetting. The cell suspension was centrifuged at 400g for 4 min at 4 °C. The cell pellet was resuspended in 1 ml ice-cold PBS + 0.04 % BSA and filtered through a 30 μm cell strainer. Counting and viability were assessed using Trypan blue staining with a Countess automatic cell counter (Thermo Fisher Scientific).

GBM 2D monocultures were singularized by Accutase, centrifuged, resuspended in 1 ml ice-cold PBS + 0.04 % BSA and filtered through a 30 μm cell strainer.

Single cells from tumors of xenografted patient derived GBM cell lines were obtained as described in Hai et al [32].

Single cell library preparation was performed using the 10x Genomics Chromium platform according to the Chromium Single Cell 3′ Reagent Kits User Guide (v3.1 Chemistry). The prepared cDNA libraries were processed by the NGS Core Facility of the German Cancer Research Center (DKFZ). The libraries were sequenced on two lanes on the Illumina HiSeq 4 K platform with a protocol specific for 10x scRNA libraries (paired-end 26 + 74).

### Single cell RNA data processing

Raw sequencing data (fastq-files) were imported in cellranger (10x Genomics) followed by preprocessing and quality control (QC) of the resulting count matrices using the R package Seurat v.4.3.0 [33]. Preprocessing, QC, and downstream analyses were performed individually for experiments in each GBM cell line (S24 and P3). First, features that were not expressed in at least one cell were removed from the count matrix. Next, QC was performed individually for each dataset from the different experimental conditions based on the distribution of feature expression, UMI counts, and mitochondrial gene expression. QC parameters are displayed in Supplementary Table S1. After exclusion of cells that did not pass the QC thresholds, Seurat objects were generated and raw counts were normalized using *NormalizeData*. Individual Seurat objects from the different experimental conditions were merged and dimensionality reduction was performed using *RunPCA* and *RunUMAP*. Datasets were then integrated using harmony v.0.1.1 based on the first 20 principal components and a maximum number of 50 clustering-correction steps (max.iter.harmony = 50). Clusters were identified using Seurat́s *FindClusters* function with a resolution threshold of 0.2. To separate malignant (i.e. GBM cells) from non-malignant cells (i.e. neurons) in the single cell analysis, CNV calling was performed using inferCNV v.1.14.2 (https://github.com/broadinstitute/inferCNV). CNV calling confirmed cluster formation based on malignant/non-malignant cell status. Using *FindAllMarkers* the top 100 markers for GBM cells and neurons dependent on the experimental condition were identified and an expression heatmap was generated using *DoHeatmap* to identify trends in gene expression changes for top marker genes of the cellular background (malignant/neuronal cells). To investigate the maturity of the cortical spheroid neurons used in the present study, we compared transcriptional states between cortical spheroid neurons and neurons of different maturation states from the same genetic background by performing comparative analyses with data from Wilkens et al. [34]. Integration of the scRNAseq datasets was performed using harmony v.0.1.1, followed by annotation according to clusters 1–6 from Wilkens et al. [34]. A pseudotime trajectory was calculated in the integrated dataset using monocle3 with the starting point in the immature neuron cluster (cluster 1 from Wilkens et al. [34]). To further investigate neuronal maturity, we performed neuron maturity index scoring using the neuMatIdx package [35]. Discriminating neuron maturity index (dNMI) and neuron functionality index (NFI) scores were visualized using violin plots and comparisons were performed across clusters. Significance testing was performed using one-way ANOVA and a Tukey HSD post-hoc test.

### GBM cell identity scoring

Using curated lists of gene expression signatures for GBM meta modules from Neftel et al [6], cell identity scoring was performed using the *AddModuleScore* function in Seurat. GBM cells were assigned to NPC-like1 (neural precursor cell-like1), NPC-like2, OPC-like (oligodendrocyte precursor cell-like), AC-like (astrocyte-like), MES-like1 (mesenchymal cell-like1), and MES-like2 categories based on the calculated identity score. For each cell, the maximum identity score observed for a meta module was used for classification. Further, cell cycle state was inferred based on the *CellCycleScoring* function resulting in classification into G1, S, or G2M phase.

### Differential expression testing

Differentially expressed (DE) genes were identified using a Mann-Whitney-*U* test as implemented in *FindMarkers*. DE testing was restricted to genes that displayed robust expression in both of the compared conditions (min.pct = 0.25). Genes surpassing threshold parameters of Bonferroni-adjusted p-value < 0.05 were considered DE genes.

### GO enrichment analysis

Functional enrichment of DE genes within biological pathways was investigated in a GO enrichment analysis. Using the *compareCluster* function of the R package clusterProfiler v.4.6.2 [36], DE gene lists from S24 and P3 cell line comparisons were jointly analyzed and visualized. The “enrichGO” function and the org.Hs.eg.db v.3.16.0 reference were called inside compareCluster and pathways remaining statistically significant (p < 0.05) after Benjamini-Hochberg (FDR) correction were selected for visualization. Visualizations were generated using the *dotplot* and *emapplot* functions from the enrichplot package v.1.18.3. To explore the fraction of genes annotated to specific GO terms such as “glutamate receptor binding”, count percentage estimates were generated for each cell. Visualization was performed using a violin plot. To generate a percentage of counts in features estimate, normalized counts were extracted for GO-term related genes using org.Hs.eg.db v.3.16.0 followed by division of the resulting sum for pathway genes by the total number of normalized counts for each individual cell. For KEGG terms, genes were extracted using KEGGREST v.1.38.0. Comparison between conditions was performed using a Mann-Whitney-*U* test for two-group comparisons and a one-way ANOVA with Tukey HSD post-hoc test for multiple-group comparisons.

### Chemo- and radiotherapy

For combinatorial chemo- and radiotherapy treatments, after 9–10 days of hGliCS culture, the hGliCS were treated with 2 x 2 Gy radiotherapy (MultiRad Faxitron 225) on two consecutive days. The radiotherapy regimen was determined before to be a dose effective against GBM cells in hGliCS in a similar manner as in patients with a limited effect and limited toxicity on neuronal cells. Drug treatments with 200 µM temozolomide (Sigma Aldrich, T2577), 30 µM lomustine (Sigma Aldrich, L5918), drug A (CK2 inhibitor), or drug B (HDAC inhibitor; all drugs in DMSO), started on day 12 of hGliCS culture and lasted for 14 days with medium changes every other day. After treatment the hGliCS were cultured for 2–3 more days. Then, they were fixed and processed for immunocytochemistry stainings. All treatments were compared to hGliCS cultured with DMSO as mock treatment. All chemo- and radiotherapy experiments were performed with cortical spheroids derived from hiPSC line CIMHi001-A and with GBM lines S24 and P3.

### Statistical analyses and visualization

**Fig. 1:**
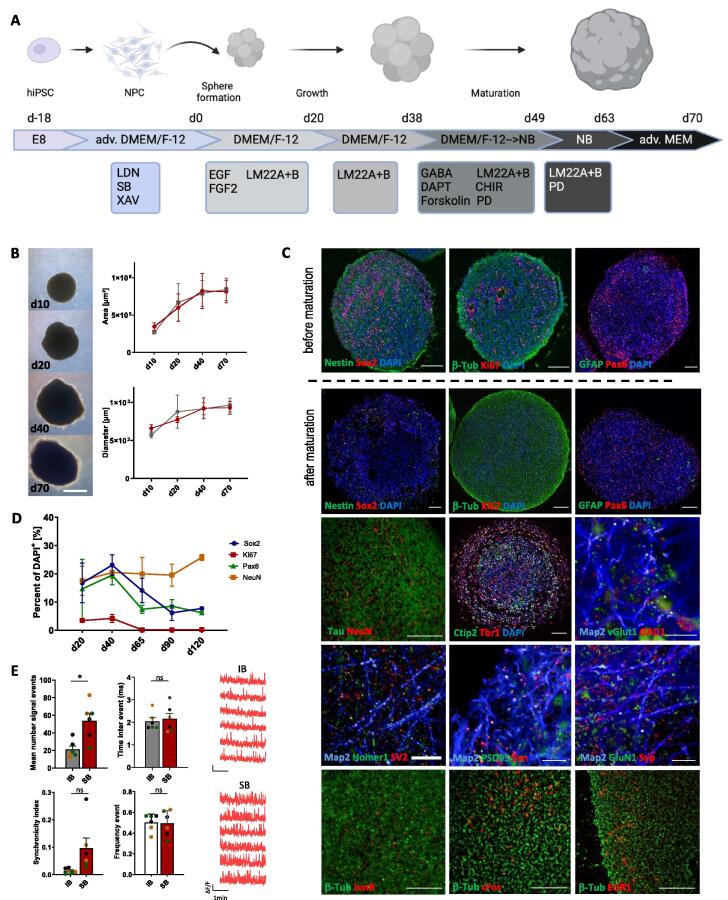
**Development of a human cortical spheroid model system. (A)** Schematic outline of cortical spheroid generation from hiPSC. The neural induction to NPC by dual SMAD inhibition was performed in 2D prior to formation of 3D neurospheres (d0). Spheroids grew in size under growth factor-enriched conditions and small molecule intervention promoted terminal differentiation to mature cortical spheroids. **(B)** Representative brightfield images of cortical spheroids during differentiation phases and size measurements of area and diameter of spheroids derived from two hiPSC-lines (displayed in red and grey, respectively). N ≥ 10 spheroids were measured per line per time point. Displayed are mean ± SD. Scale bar: 500 µm **(C)** Representative immunofluorescences of precursor marker proteins Sox2, Nestin, Pax6, proliferation marker Ki67, and astrocytic marker GFAP before (upper row) and after (second row) maturation phase of differentiation. Third row: Expression of neuronal maturation marker NeuN, cortical layer markers Tbr1 and Ctip2, excitatory marker vGlut1 and inhibitory GABAergic marker GAD1. Fourth row: Expression of pre- and postsynaptic markers SV2, Syn, Syp, Homer1, PSD95, and GluN1, respectively. Lowest row: Expression of immediate early genes JunB, cFos, and EGR1. Scale bars: 100 µm, (except for vGlut1/GAD1 and synaptic markers: 10 µm). **(D)** Quantification of nuclear markers Sox2, Pax6, Ki67, NeuN during differentiation (N = 3 Spheroids, from n = 3 spheroid batches). Displayed are mean ± SD. **(E)** Calcium imaging data displaying functionality of neurons. Recordings were performed in imaging buffer (IB; without potassium) and stimulation buffer (SB; with 8 mM potassium). Neurons were responsive and capable of generating calcium signals in response to stimulation. Graphs show mean number of signal events (upper left), time inter events (upper right), synchronicity index (lower left), and frequency events (lower right). Data are displayed as mean ± SEM (N = 6 with data points from n = 3 spheroid batches (colored in blue, green, orange); per spheroid batch N = 2 spheroids were used with N = 70–100 analyzed ROIs per recording); Mann-Whitney-*U* test for independent samples was calculated; *p < 0.05. On the right, typical Ca^2+^ transients for each condition (IB + SB).

**Fig. 2:**
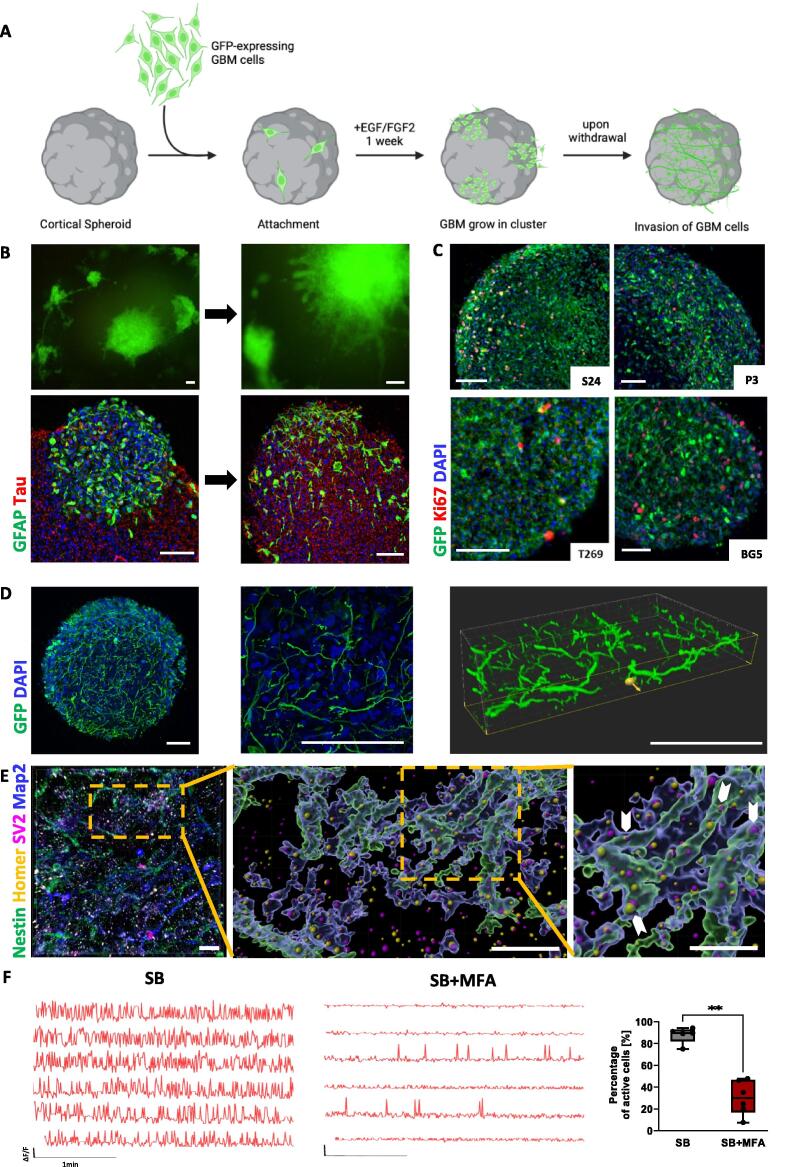
**Infiltration of cortical spheroids by GBM tumor cells reflect tumor key hallmark features. (A)** Schematic outline of the hGliCS model. GFP-expressing GBM cells were added to cortical spheroids as single cells. In the presence of EGF/FGF2, GBM cells grew in clusters and only upon withdrawal of growth factors GBM cells invaded cortical spheroids. **(B)** Representative *in vitro* and immunofluorescence images of hGliCS with GBM line S24 before (day 7 of hGliCS culture) and after EGF/FGF2 withdrawal (day 10) showing invasion of GBM cells into the cortical spheroid. **(C)** Representative images of four different GBM cell lines, S24, P3, BG5, T269, after 4 weeks of hGliCS culture showing fully colonized cortical spheroids for all four GBM lines. **(D)** High-resolution imaging of hGliCS with P3-GFP after 4 weeks. Left: Overview displaying a complete spheroid (20x objective), Middle: Maximum projection of a zoom section (63x objective), Right: 3D-Imaris reconstruction of the z-stack of a zoom section shows microtube formation of invaded GBM cells. **(E)** Confocal image of hGliCS culture with S24 after 4 weeks. Multiplexing with spectral separation was performed for GBM marker Nestin (green), Map2-positive neuronal cells (blue), presynaptic marker SV2 (purple), and postsynaptic marker Homer1 (yellow). The areas for Nestin and Map2, as well as the spots for SV2 and Homer1 were reconstructed with Imaris (middle and right image). Many Homer1-spots can be detected on tumor cells and colocalize with SV2 spots which are located on neurons pointing to potential neuron-glioma-synapses (indicated by white arrows in right picture). **(F)** Typical Ca^2+^ transients of S24GCaMP GBM cells after 7 weeks of hGliCS culture before and after application of 100 µM gap junction inhibitor MFA recorded in stimulation buffer (SB; with 8 mM potassium). The graph shows the percentage of active cells before and after MFA application. Displayed are mean ± SEM from three independent experiments with n = 240 cells per condition analyzed; Mann-Whitney-*U* test for independent samples was calculated; **p < 0.01. Scale bars: 100 µm, in **(E)**: 10 µm.

**Fig. 3:**
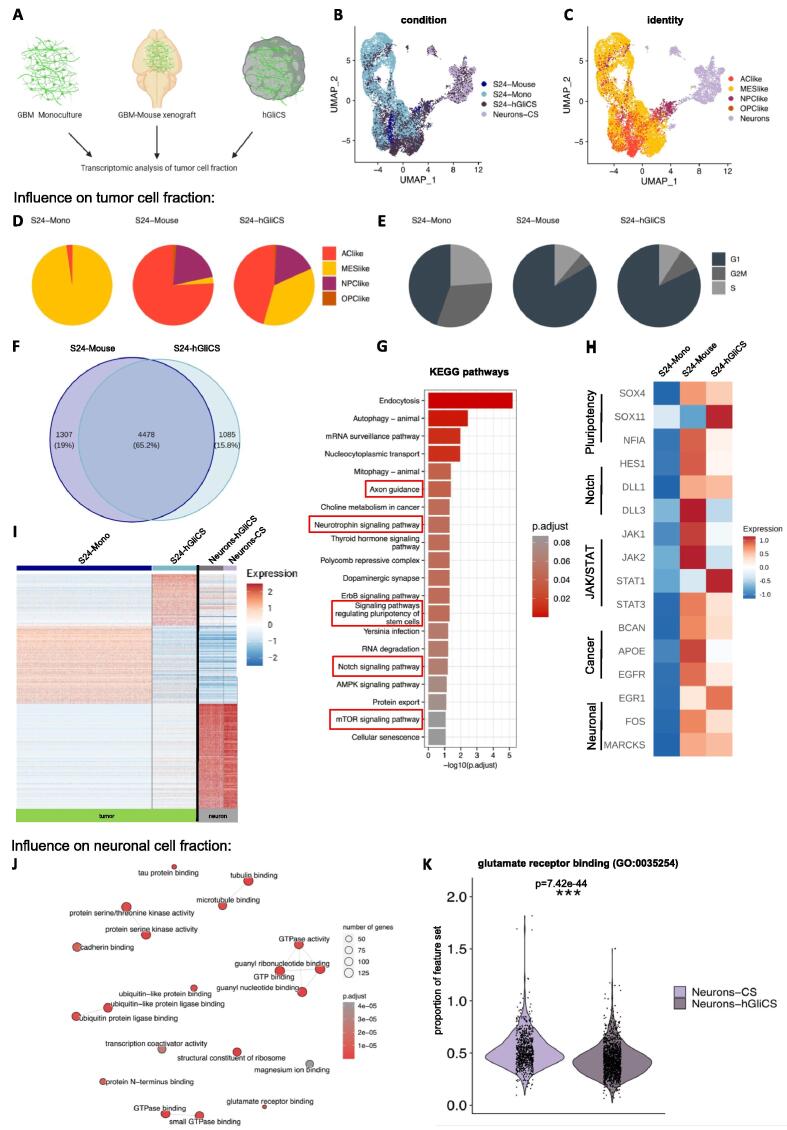
**Single cell transcriptomic analyses of environment-dependent models reveal high congruency between hGliCS and xenograft. (A)** Schematic overview of model generation for scRNAseq experiments. In (D) – (H) only the GBM cell fraction from the respective conditions were analyzed (termed S24-Mono, S24-Mouse, and S24-hGliCS). In (J) and (K) only the neuronal fraction of S24-hGliCS compared to cortical spheroids without GBM cells were analyzed (termed Neurons-hGliCS and Neurons-CS). **(B)** UMAP projection of S24 GBM cell fraction across all models and the neuronal fraction from cortical spheroids (Neurons-CS) displaying significant overlap of the three models and at the same time a clear separation between neurons and GBM cells. **(C)** UMAP projection of S24 GBM cell fraction across all models according to GBM subtypes based on Neftel gene sets [6] and the neurons from cortical spheroids (AClike: astrocyte-like, MESlike: mesenchymal-like, NPClike: neural progenitor cell-like, OPClike: oligodendrocyte progenitor-like). **(D)** Pie charts depicting percentage of GBM subtype based on Neftel gene sets [6] in S24 GBM cell fraction by model type. S24-Mono primarily consisted of MES-like cells, S24-Mouse predominantly of AC-like cells. S24-hGliCS displayed increased cell state diversity compared to S24-Mono. **(E)** Pie charts reflecting the distribution of cell cycle state in S24 GBM cell fraction by model type. S24-Mono displayed a higher fraction of cycling cells compared to both, S24-hGliCS and S24-Mouse. **(F)** Venn diagram of upregulated DEGs in S24-hGliCS and S24-Mouse (compared to S24-Monoculture) displaying strong overlap between DEGs. **(G)** Bar plot showing the top 20 terms of a KEGG pathway analysis of the shared upregulated DEGs (compared to S24-Mono) from Fig. 3F sorted by adjusted p-value. Relevant neuron- or GBM-related terms are highlighted. **(H)** Heatmap of scaled and centered expression values for genes selected based on KEGG analysis or literature. While comparable expression levels were found between S24-hGliCS and S24-Mouse conditions, strong differences were observed compared to the S24-Mono condition. **(I)** Heatmap of top 100 marker genes of the GBM fraction in hGliCS (left part, highlighted in green) and the neuronal fraction in hGliCS (right part, highlighted in grey) compared to the respective monoculture (S24-Mono for GBM fraction and Neuron-CS for neuron fraction). Scaled and centered gene expression levels are displayed in the heatmap. **(J)** Enrichment map showing top 20 most enriched GO biological processes in the neuronal fraction of the S24-hGliCS model. The color reflects the p-adjust value and the size of the dot the number of contributing genes. Neuron-specific terms like tubulin-binding, tau protein-binding, or glutamate receptor binding were enriched. **(K)** Violinplot showing expression of genes contributing to GO term glutamate receptor binding in the neuronal fraction of hGliCS compared to Neuron-CS (without GBM cells). Genes associated with the GO term glutamate receptor binding were down-regulated in hGliCS. For a comparison of the proportion of pathway gene counts in the total number of counts a Mann-Whitney-*U* test for independent samples was calculated; ***p < 0.001. For (F) – (J) detailed statistical procedures are described in Materials and Methods.

Unless stated otherwise, all results are displayed as means with standard error of the mean (SEM). Graphs were generated using GraphPad Prism 10.1.2, Excel (Fig. 1 B + D), and the R statistical computing environment v.4.2.1. For statistical analysis GraphPad Prism 10.1.2 was used. Prior to statistical analysis data was checked for normal distribution with Shapiro-Wilk normality test. Multiple groups were compared with parametric One-way ANOVA. If only two groups were compared a non-parametric Mann-Whitney-*U* test for independent samples was performed. Significance levels against the respective controls used are *p < 0.05, **p < 0.01, ***p < 0.001, ****p < 0.0001 and ns: not significant (p > 0.05). Schematic representations in Fig. 1, Fig. 2, Fig. 3 were generated using biorender.com.

## Results

### Development of a human glioblastoma-in-brain-spheroid model

To establish the hGliCS model, neuronal precursor cells of dorsal cortical identity generated from hiPSCs were first aggregated to form cortical spheroids (illustrated in Fig. 1A). Aggregates were expanded for 20 days in the presence of the growth factors FGF2 and EGF. This was followed by a neuronal differentiation and maturation phase of 18 days which included the TrkB/TrkC receptor antagonists LM22A and B. From day 38, maturation and differentiation of the neurons was further accelerated by adding the Notch inhibitor DAPT, the GSK3β inhibitor CHIR99021, the cyclin inhibitor PD-0332991, as well as Forskolin and GABA. The morphological development of the cortical spheroids during these stages is exemplified in Fig. 1B. Growth measurements have been assessed for spheroids derived from two independent hiPSC lines (Fig. 1B). Immunocytochemical staining of typical marker proteins confirmed the characteristics of the cortical spheroids (Fig. 1C): After the expansion phase and before neurons started to mature, spheroids consisted of a mixture of neural progenitors (positive for Sox2, Pax6, and Nestin; upper row) and neurons (expressing β-III-tubulin). Proliferation of the neural progenitor fraction was indicated by the presence of the proliferation-associated antigen Ki-67. Glial fibrillary acid protein (GFAP) expressing astrocytes were absent at this stage. At the end of the maturation stage, expression of Nestin, Sox2 and Ki-67 was detected only occasionally. Instead, neurons became dominant showing expression of NeuN. Most neurons expressed the transcription factors Tbr1 and/or Ctip2 indicating a layer 5/6 cortical identity. In line with this, vGlut1 (glutamatergic neurons) was present on most of the neurites and GAD1 positive cells (GABAergic neurons) were the minority. Few GFAP-positive astrocytes were present at this stage. Neurons also expressed pre- and postsynaptic marker proteins such as SV2, synapsin (Syn), synaptophysin (Syp), and Homer1, PSD95 or GluN1. Expression of immediate early genes such as cFOS, JunB or EGR1 were detected indicating the development of functional neuronal networks.

To evaluate neuronal maturity, we performed a comparative analysis using scRNAseq data. We analyzed data from our cortical spheroids alongside the neuronal maturation dataset published by Wilkens et al. [34]. Their study provided valuable reference data by analyzing neurons of matching genetic background across multiple developmental stages. Through this comparison, our analysis revealed that neurons derived from our cortical spheroids exhibited maturity markers that surpassed even the most advanced developmental stages documented in the Wilkens et al. study (Fig. 1S).

To enhance the interaction between neurons and GBM tumor cells we further implemented a strategy to activate the neurons. This was achieved by changing the base medium from Neurobasal to advanced MEM which provides an improved physiological condition comparable to Brainphys as described by Bardy et al [37]. Functional properties of the spheroids were assessed by Ca^2+^-imaging experiments. We observed Ca^2+^-transients which increased in number upon stimulation with high potassium (8 mM). Stimulating conditions additionally increased their synchronicity (Fig. 1E). This indicates that the neurons within the spheroids were responsive and capable of generating calcium signals in response to stimulation. Overall, these results demonstrate a successful generation of human cortical spheroids with characteristics resembling mature neurons which was a significant prerequisite to study GBM biology in a complex brain model.

### Morphological and functional properties of infiltrated GBM tumor cells in cortical spheroids reflect key glioma hallmarks

The hGliCS culture model was established as depicted in Fig. 2A. GBM cells were suspended to single cells and 5000 cells were added per cortical spheroid. Initially, GFP-expressing GBM cells attached to the surface of cortical spheroids as single cells. In the presence of EGF and FGF2 GBM cells grew in clusters that were still located largely attached to the spheroid as shown by live-cell imaging and by immunofluorescence staining against GFAP and neuron specific marker Tau in Fig. 2B (left side). The content of endogenous GFAP expressing cells in the cortical spheroids is neglectable at this stage and GFAP can therefore be considered as marker for GBM cells in this setting (compare to Fig. 1C). After one week of culturing, withdrawal of the growth factors EGF and FGF2 resulted in resolving of GBM clusters, and within 3 days, the cells started to invade the spheroid (Fig. 2B, right side). This growth factor withdrawal was crucial for GBM cell invasion; the initiation of infiltration into the cortical spheroid platform and glioma colonization per se was demonstrated using four different patient-derived GBM tumor lines, S24, P3, BG5, and T269 (Fig. 2C).

GBM cell connectivity and communication have been described to critically contribute to malignancy and resistance [9,10,[21], [22], [23], [24],^38^]. Therefore, we aimed to recapitulate these hallmark features in our hGliCS model. One of these features are characteristic, long membrane protrusions, called tumor microtubes (TMs), that are drivers of the diffuse infiltration of GBM cells in the brain in patients and in murine models [22]. Furthermore, TMs contribute to GBM cell proliferation [22,39], and interconnect single GBM cells to one communicating network which is resistant against standard therapies like radiation or chemotherapy, and able to repair itself [22,40,41]. The presence of TMs in the hGliCS model was demonstrated by immunofluorescent staining against GFP (expressed exclusively by GBM cells). A 3D-reconstruction of the GFP signal revealed that TMs built a dense network of GBM cells similar to the human disease (Fig. 2D).

A second feature of cancer-intrinsic neural mechanisms that drives glioblastoma malignancy is the GBM cell integration into existing neuronal networks, including in patients, via the formation of glutamatergic synapses from neurons onto GBM cells. In these synapses, GBM cells are the post-synaptic partner expressing AMPA receptors, with most of the synapses located on TMs. [9,10]. This interaction has been demonstrated to promote tumor proliferation and invasion [9,25]. In our hGliCS model potential neuron-glioma-synapses were observed by multiplexing with spectral separation on immunofluorescent stainings against the presynaptic marker SV2 and postsynaptic marker Homer1 (Fig. 2E). In a reproduction of Nestin and Map2 areas, as well as a spot detection of SV2 and Homer1 using Imaris, Homer1 signals (yellow) were clearly visible on Nestin expressing GBM cells (green). When their TMs were in close proximity to Map2-expressing neurons (blue) many colocalizing events of Homer1 and SV2 (purple) were observed pointing to potential synapses.

A third neural feature of gliomas is the communication of interconnected GBM cells via calcium transients [24]. The spread of network transients from one GBM cell to another along TMs relies on gap junctions, formed by gap junction protein Cx43 [22]. Experimentally, we could demonstrate the existence of gap junctions in the hGliCS model through the application of the gap junction inhibitor meclofenamic acid (MFA), which led to the decrease of active GBM cells as it was shown before [22,24,42] (Fig. 2F). The small proportion of actively remaining GBM cells might indicate that some cells escaped gap junction triggered activity and exhibited intrinsic autonomous calcium transients as recently described [24].

### Cellular states of GBM tumor cells invaded in cortical spheroids are comparable to *in vivo* mouse xenografts

GBM cell lines have a characteristic cellular signature and their migratorial behavior is significantly influenced by microenvironment-associated transcriptomic plasticity [6,43,44]. To examine the cell state composition within the GBM compartment, scRNAseq experiments were conducted on two monocultured GBM lines, a cortical spheroid culture and from two hGliCS cultures. The findings were compared with those obtained from mouse xenografts of the same GBM lines. Additionally, based on the single-cell data a detailed analysis was performed to examine the reciprocal influence between GBM cells and neurons in hGliCS by clustering analysis in comparison to their respective monocultures. The analyzed models are schematically represented in Fig. 3A, with mouse xenografts being excised 90 days post GBM injection and hGliCS cultured with GBM cells for 28 days, which both allow sufficient GBM establishment representative of the human disease. In order to ensure analysis of malignant cells, these cells were classified by a previously defined copy number variation (CNV) analysis and non-malignant cells corresponding to neuronal cell clusters (Figs. S2 for S24 and S3 for P3).

In a first step, we analyzed the impact on the GBM cell fraction in all conditions (termed S24-Mono, S24-Mouse, and S24-hGliCS; for P3 accordingly). The UMAP projection for the primary GBM cell culture S24 in Fig. 3B exhibited significant overlap for the three models (P3 in Fig. S4A). GBM cells clearly separated from neuronal cells (Neurons-CS). GBM cell state assignments, established in Neftel et al [6], were plotted in an UMAP projection (Fig. 3C; result for GBM cell line P3 in Fig. S4B). Notably, in a comparison of the GBM cell fraction in all three models monocultured GBM cells (in a 2D monolayer) primarily consisted of 97.6 % MES-like cells, while the AC-like subtype dominated in the mouse xenografts (75.8 %). The hGliCS model demonstrated increased cell state diversity compared to the GBM monoculture condition, as depicted in the pie charts in Fig. 3D (for P3 in Fig. S4C). A cell cycle analysis revealed a higher fraction of cycling cells in monocultured GBM cells, while both, the hGliCS model and the mouse xenograft showed a comparable reduction of cycling cells (Fig. 3E; result for P3 in Fig. S4D).

### Differential expression analysis highlights similarities between mouse xenograft and hGliCS

In a differential gene expression analysis we initially examined the overlap of differentially expressed genes (DEGs) within the GBM cell fraction between mouse xenografts and hGliCS, both in comparison to monocultured GBM cells (in 2D layer). About 65 % of DEGs in both analyses were overlapping, as depicted in the Venn diagram in Fig. 3F. To attribute specific biological processes and pathways affected by different tumor microenvironments within this subset we conducted KEGG and gene ontology (GO) pathway analyses. In S24-hGliCS, the top-scoring pathway of the KEGG ontology was endocytosis, followed by autophagy, indicating a modulation of vesicle-related pathways. Pathways related to neuronal function such as axon guidance or neurotrophin signaling were among the top 20 upregulated KEGG pathways (Fig. 3G). Several other enriched pathways were suggestive of stem cell-like characteristics of malignant cells, such as the pluripotency pathway (including genes like *SOX4, SOX11*, and *NFIA*), and Notch signaling pathways (including genes like *HES1, DLL1,* and *DLL3*). Additionally, the mTOR pathway indicating a link to the JAK/STAT pathway (including genes like *JAK1, JAK2, STAT1,* and *STAT3*) which has been described in the context of protumorigenic functions [43]. A combined GO analysis for both tumor lines (S24 + P3) in both models (hGliCS + mouse) is shown in Fig. S4E, revealing shared cell homeostasis related terms (ATP-dependent activity, cadherin binding, GTPase, NADH, ubiquitin binding) across all four conditions. For a further examination of individual genes, we selected genes from the enriched tumor- or neuronal function-related KEGG pathways (Fig. 3G) and published gene sets (as f. ex., typical tumor drivers *BCAN, APOE*, and *EGFR* or genes associated to neuronal function like *EGR1, FOS*, and *MARCKS*) [[45], [46], [47]]. The heatmap in Fig. 3H illustrates the gene expression levels of these genes in the S24 tumor cell fraction across all three models (result for P3 in Fig. S4F). Stemness-related genes *SOX4* and *NFIA* exhibited expression in both, mouse xenograft and hGliCS, with *SOX11* showing high expression exclusively in hGliCS, compared to monocultured S24 tumor cells. Notch genes *HES1* and *DLL1* were robustly expressed in both models, while *DLL3* exhibited a specific expression in the mouse xenograft. JAK/STAT pathway related genes *JAK1* and *JAK2* were stronger expressed in the mouse xenograft, *STAT1* in the hGliCS model, and *STAT3* in both models. Tumor drivers like *BCAN, APOE*, or *EGFR* were stronger expressed in both models compared to monocultured GBM cells, as were genes associated with neuronal function, including *EGR1, FOS,* or *MARCKS.* A comparison of gene expression levels for complete KEGG pathways for all three models is displayed in Fig. S5 showing a higher similarity between hGliCS and mouse xenograft compared to monocultured GBM. Taken together, these results suggest a modulation of concordant pathways in the tumor cells in both, the hGliCS model and the mouse xenograft condition.

### Influence of tumor cells on neural microenvironment

To assess the impact of tumor cells on neurons, we performed in a next step a DEG analysis for the hGliCS model in comparison to the respective single-cell data set from cortical spheroids without tumor cell infiltration. The heatmap in Fig. 3I displays unique marker genes specific for each of the conditions (S24-Mono, S24-hGliCS, Neurons-hGliCS, Neurons-CS). While the tumor cells exhibited significant alterations in their expression profiles due to the influence of the neuronal microenvironment (described in the last paragraph and shown in Fig. 3G,H), the neurons themselves showed only subtle changes when compared to their counterparts in tumor-free cortical spheroids of the same age. Interestingly, we see in the neurons of the hGliCS model a downregulation of many structural genes, like *TUBB3*, *MAPT*, *MAP2* (Fig. S6). At the same time, the GO analysis of DEGs in the neuronal fraction of hGliCS unveiled biological process terms such as tubulin binding, tau protein binding, cadherin binding and, notably, magnesium ion binding and glutamate receptor binding (Fig. 3J). An in-depth analysis of the term glutamate receptor binding revealed a coordinated down-regulation of genes associated with this pathway in the hGliCS model (Fig. 3K).

### hGliCS as model system for GBM tumor treatment

**Fig. 4:**
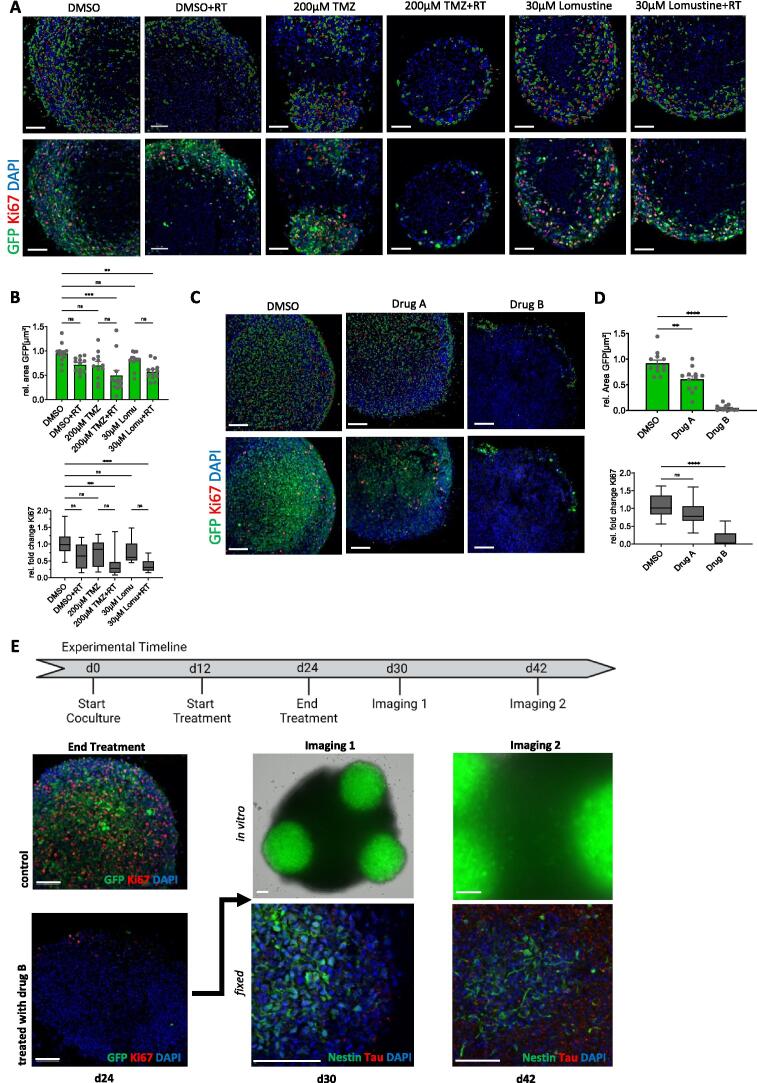
**HGliCS as model system for GBM tumor treatment. (A)** Treatment of hGliCS (with GBM line P3) with classical cytostatic drugs TMZ or Lomustine in comparison to controls (DMSO), all +/- combination with radiotherapy (RT). The upper row of pictures displays the Imaris reconstructions which were acquired on the basis of the original immunofluorescences (lower row). **(B)** Quantifications of tumor area reflected by GFP expression and proportion of Ki67-positive cells based on (A) show a significant cytotoxic treatment effect only in combination with RT (Graphs show means ± SEM from n = 2 experiments, with N = 6 spheroids per experiment). Each treatment was compared to control (DMSO) and to the respective condition without RT. **(C)** Treatment of hGliCS (with GBM line S24) with explorative anti-TM-drugs in comparison to controls (DMSO). The pictures show in the upper row Imaris reconstructions acquired on the basis of the immunofluorescences in the lower row. **(D)** Quantifications of tumor area and proportion of proliferative cells based on Imaris reconstructions of GFP and Ki67 stainings. (Graphs show means ± SEM from n = 2 experiments, with N = 6 spheroids per experiment). The effect of drug A is moderate cytotoxic and not significant on cell proliferation. Drug B shows a strong effect on cell viability and proliferation. Each treatment was compared to control (DMSO). **(E)** Recurrent tumor growth after a 2 week-treatment with anti-TM-drug B (maximal effect on day 24 of hGliCS culture (with GBM line S24) in comparison to control (DMSO), upper and lower left). Already 6 days later small clusters of GBM cells were visible which started to invade the spheroid after further 12 days of culturing. Upper pictures (middle and right) show *in vitro* conditions, lower pictures (middle and right) show immunofluorescences against GBM marker Nestin and neuronal marker Tau. For all quantifications multiple groups were compared by calculating One-way ANOVA with Tukey HSD post-hoc test. Significance levels against the respective control: *p < 0.05, **p < 0.01, ***p < 0.001, ****p < 0.0001 and ns: not significant (p > 0.05).

In order to validate the hGliCS model for drug screening tests we first treated hGliCS with classical cytostatic drugs commonly used in clinical GBM contexts [1,48,49]. Specifically, following invasion and formation of the GBM network we applied 200 µM Temozolomide (TMZ) or 30 µM Lomustine either as monotherapy or in combination with radiotherapy (RT) compared to controls +/- RT (Fig. 4A + B). The treatment effect was evaluated in Imaris reconstructions (Fig. 4A, upper row) from immunofluorescences (Fig. 4A, lower row) via quantification of the GFP signal expressed in S24 GBM cells (Fig. 4B, upper graph), and by quantification of the fraction of Ki67-positive cells (Fig. 4B, lower graph). Both analyses clearly demonstrated a visible, although not significant, RT effect when applied as a single treatment and a cytotoxic effect on the GBM cells only when combined with RT. This is in line with previous observations of a high resistance of GBM stem-like cell lines like S24 towards chemo- and radiotherapy through network integration of GBM cells [40].

To overcome network resistance and to discover and develop new treatment strategies, we tested the effect of exploratory anti-TM-drugs. We used a casein kinase 2 (CK2) inhibitor (drug A) and a histone deacetylase (HDAC) inhibitor (drug B) on hGliCS (Fig. 4C; Imaris reconstruction in upper row, original immunofluorescence in lower row). The inhibition of CK2 is based on its overexpression in GBM and its role in phosphorylating GAP43 in growth cones. Research has shown that suppressing CK2 activity effectively reduces GBM growth [50]. The second target, HDACs are also overexpressed in GBM. HDAC inhibitors have demonstrated multiple beneficial effects: they interfere with glioma cell protrusion, downregulate Cx43, and reduce calcium signaling activity within glioma networks [51]. Drug A showed a moderate cytoreductive effect (GFP area quantification) while the influence on proliferation was not significant (Ki67 quantification; Fig. 4D). GFP area and Ki67 quantifications (Fig. 4D), showed a strong effect of drug B on both, GBM cell area and proliferation of the GBM cells. Notably, no significant cell death could be detected in the cortical spheroids per se after exposure to cytostatic drugs (Fig. S7).

Interestingly, hGliCS treated with drug B for 14 days started to develop new GBM clusters within 6 days likely arising from single residual GBM cells (Fig. 4E). After further culturing for 12 days, these GBM clusters resolved and infiltrated the spheroid with the formation of new TMs like the original GBM morphology (Fig. 4 E, stained by Nestin in the lower right picture). This resembles tumor relapse from resistant clones, similar to the situation observed in patients and can thus pose as a new model to study treatment effects, residual disease and recurrence longitudinally.

## Discussion

Recent research on GBM has uncovered two key features that link GBM with neuron-like behavior. GBM cells form structures similar to neurites (extensions from neurons), show invasion patterns akin to those seen in neurons [21], and establish complex networks that include rhythmic calcium signaling, controlled by cells that act like pacemakers [24]. The second key feature is the direct interaction between GBM cells and neurons via specialized synapses, which facilitate tumor growth and invasion [9,10,21,52,53]. These findings emphasize how the neuronal environment plays a critical role in the development of GBM and show the need for advanced experimental models to study it more effectively.

Three-D modeling of GBM has significantly improved how we understand tumor biology. By growing GBM stem cells alongside human organoids, researchers can better simulate how tumors behave in a more biologically accurate setting [15,16,19,[54], [55], [56]]. These models let researchers closely study tumor growth and how tumors invade. They use patient-derived GSCs in brain organoids to replicate the characteristics of human GBM, maintaining both gene expression and cellular interactions that are crucial for GBM's aggressive nature.

Although the new models are promising, they still have challenges. One issue is the variability within cerebral organoids, especially with progenitor cells, which could complicate the interpretation of results and limit their applicability to real-world scenarios. Progenitor cells within these organoids may affect the behavior of the tumor, creating complexities that make it harder to directly apply findings to real-life situations.

The hGliCS model overcomes some of these issues by offering a more mature, functional human brain environment, where neuronal differentiation happens in a short time.

In the hGliCS model, GBM cells exhibit three key features observed in patient tumors. One of these is the development of extensive networks, called TMs, which contribute to the tumor’s resistance to treatment. Secondly, the GBM cells form synaptic connections with neurons, similar to those found in living organisms [9,10]. The third feature is the formation of an interconnected network with gap junctions, allowing communication between cells and exhibiting pacemaker-like behavior [24]. This model closely replicates the neurobiological features of GBM found in patients. Gliomas, including GBMs, are highly heterogeneous due to the plasticity of the tumor, influenced by its environment [45]. The scRNAseq analysis shows that the hGliCS model accurately replicates this heterogeneity, similar to mouse xenograft models. The overlap in gene expression between the hGliCS and mouse models suggests that similar signaling pathways control how the tumor interacts with its environment in both models.

The presence of endocytosis-related pathways suggests that vesicular communication between tumor and non-tumor cells is an important mechanism for tumor-microenvironment interactions [19]. The model replicates the activation of critical pathways related to cancer development, including stem cell genes, Notch signaling, and JAK/STAT pathways [14,41,49,57,58] (for review see Dewdney et al [59]). It also shows the upregulation of markers associated with invasion and connectivity [9,[59], [60], [61]]. The analysis also identified the upregulation of genes involved in nervous system development and function, suggesting that GBM cells hijack neuronal signaling pathways to drive tumor growth. In summary, our results indicate that hGliCS are highly comparable to mouse models in capturing the complexity of glioma heterogeneity and microenvironmental influences.

An interesting finding was that GBM cells had a subtle effect on neuronal gene expression. Neurons retained their identity but showed slight downregulation of key genes involved in structural processes such as tubulin-binding and tau protein-binding. These processes are critical for axonal growth and maintenance [62] and their downregulation could help facilitate the interaction between neurons and the GBM network. The study also observed that glutamate receptor binding was downregulated in the hGliCS-neurons compared to control neurons, suggesting a change in neuronal function when interacting with GBM cells.

The reduction of glutamate receptor-related genes may be a protective response to prevent damage from excess glutamate produced by the tumor [63,64], while glutamate may also aid tumor growth by acting as a growth factor for GBM cells.

The utility of the hGliCS model was demonstrated in drug screening tests. The GBM cells within this model showed resistance to treatment but were vulnerable when combined with radiotherapy (RT). In further testing with experimental drugs, the model showed strong cytotoxic effects, and, importantly, tumor relapse occurred after treatment, mirroring how tumors relapse in patients. This observation opens the door for additional studies to understand how resistance works. It also shows the potential of this model for testing drugs in both initial treatments and relapse scenarios, which could lead to personalized treatment strategies.

Despite its significant advancements, the hGliCS model has limitations, particularly in its cellular complexity when compared to the full complexity of living organisms. While our hGliCS model successfully recapitulates key neuron-glioma interactions, we acknowledge important limitations regarding cellular complexity. The absence of microglia—brain-resident immune cells that can both suppress and promote GBM progression through cytokine signaling and phagocytosis—likely impacts inflammatory responses and immune-mediated tumor modulation in our system. Similarly, the lack of endothelial cells eliminates vascular components critical for tumor angiogenesis, blood–brain barrier interactions, and delivery of therapeutics. These limitations may affect observed drug responses, particularly for compounds targeting immune checkpoints or angiogenesis. Future iterations of our model will incorporate these cellular components through co-culture with iPSC-derived microglia and endothelial cells and improve translational relevance for drug screening applications. This expanded model would better reflect the complex tumor microenvironment and provide even more comprehensive insights into GBM biology and treatment response.

The simplicity of the model, however, also offers benefits, allowing researchers to control environmental factors for in-depth mechanistic studies. The human-specific nature of hGliCS offers advantages over animal models (xenografts), allowing for more relevant drug screening and potentially speeding up therapeutic development while reducing the need for animal testing. The ability to test both, the effectiveness of anti-tumor drugs and their neurotoxic effects in human cells may help ensure that preclinical findings translate better into successful clinical treatments [65].

In conclusion, hGliCS is a significant advancement in GBM research, bridging the gap between simplified lab models and complex living systems. The model provides a physiologically relevant human platform for studying GBM biology and testing novel therapeutic strategies that target neuron-glioma interactions, potentially accelerating the development of personalized treatments while reducing reliance on animal models that often fail to predict clinical efficacy.

## Author contributions

Sandra Horschitz, Ammar Jabali and Sophie Heuer conceptualized the project, performed experiments and analyzed data. Sandra Horschitz, Sophie Heuer, Ammar Jabali and Philipp Koch wrote the manuscript. Lea Zillich and Eric Zillich conducted processing, analysis and visualization of RNA sequencing data. Dirk C. Hoffmann and Ling Hai contributed RNA sequencing data and analyses of mouse experiments. Akshaya Senthil Kumar and Daniel Dominguez Azorin performed experiments and analyzed data. David Hausmann provided methodology. Wolfgang Wick provided resources, funding and supervised the project. Philipp Koch and Frank Winkler conceptualized the project, provided funding and supervised the project. S.Ho., A.J., S.He., L.Z., P.K., D.C.H., F.W. reviewed and edited the manuscript.

## Declaration of Generative AI and AI-assisted technologies in the writing process

During the preparation of this work the authors used Claude in order to improve readability and language. After using this tool, the authors reviewed and edited the content as needed and take full responsibility for the content of the publication.

## Declaration of competing interest

The authors declare that they have no known competing financial interests or personal relationships that could have appeared to influence the work reported in this paper.

## Data availability

The datasets generated and analyzed during the current study are available in the European Genome Archive (EGA) under accession number EGAS50000000480 (https://ega-archive.org/studies/EGAS50000000480).
