# Supplementary material for: Development of a fully human glioblastoma-in-brain-spheroid model for accelerated translational research
Source: J Adv Res. 2025 Apr 4;79:363–77. doi: 10.1016/j.jare.2025.03.055 (PMC12766203; doi:10.1016/j.jare.2025.03.055)
Supplement: Supplementary Data 3 [file mmc3.pdf]

Supplementary Figure 1

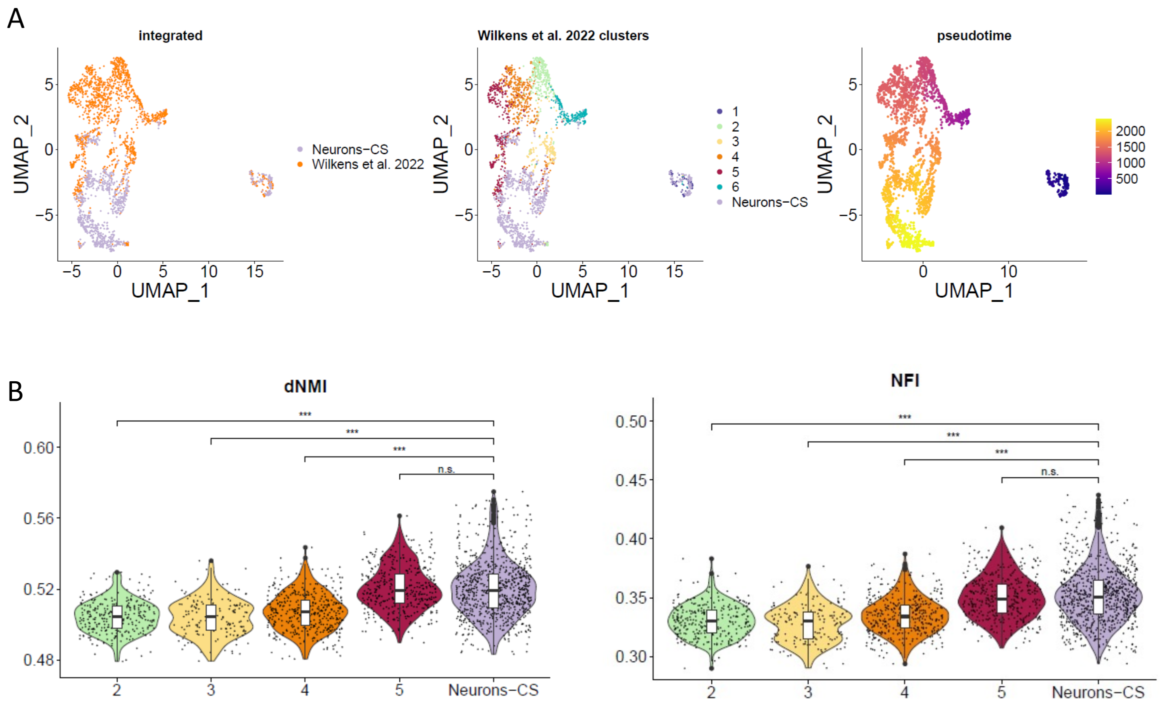

Fig. S1: Assessment of neuronal maturation state in cortical spheroids.

(A) UMAP projections of neurons from cortical spheroids (Neurons-CS) in comparison to neurons of different maturation states from the same genetic background from Wilkens et al. (34). Left panel: UMAP representation of integrated datasets. Middle panel: UMAP representation based on the annotation of cell type clusters 1-6 from Wilkens et al. (34). The neurons from cortical spheroids are located closest to the most mature population (cluster 5) from Wilkens et al. (34). Right panel: Monocle3 pseudotime analysis across Wilkens et al. clusters and neurons from cortical spheroids. Colors indicate progression in pseudotime with yellow data points marking cells that are most advanced along the trajectory. **(B)** Maturity scoring of neurons from cortical spheroids (Neurons-CS) in comparison to neuronal cell populations from Wilkens et al. (34) was performed using neuMatIdx (35). The discriminating neuron maturity index (dNMI) is calculated by focusing on transcriptional modules best explaining the differences between immature and mature neurons. The neuron functionality index (NFI) is derived from modules specifically enriched in mature neurons. One-way ANOVA for multiple groups with Tukey HSD post-hoc test was performed. Significance shows the

comparison to Neurons-CS. Significance levels: *$p < 0.05$, **$p < 0.01$, ***$p < 0.001$, ****$p < 0.0001$ and ns: not significant ($p > 0.05$).

Supplementary Figure 2

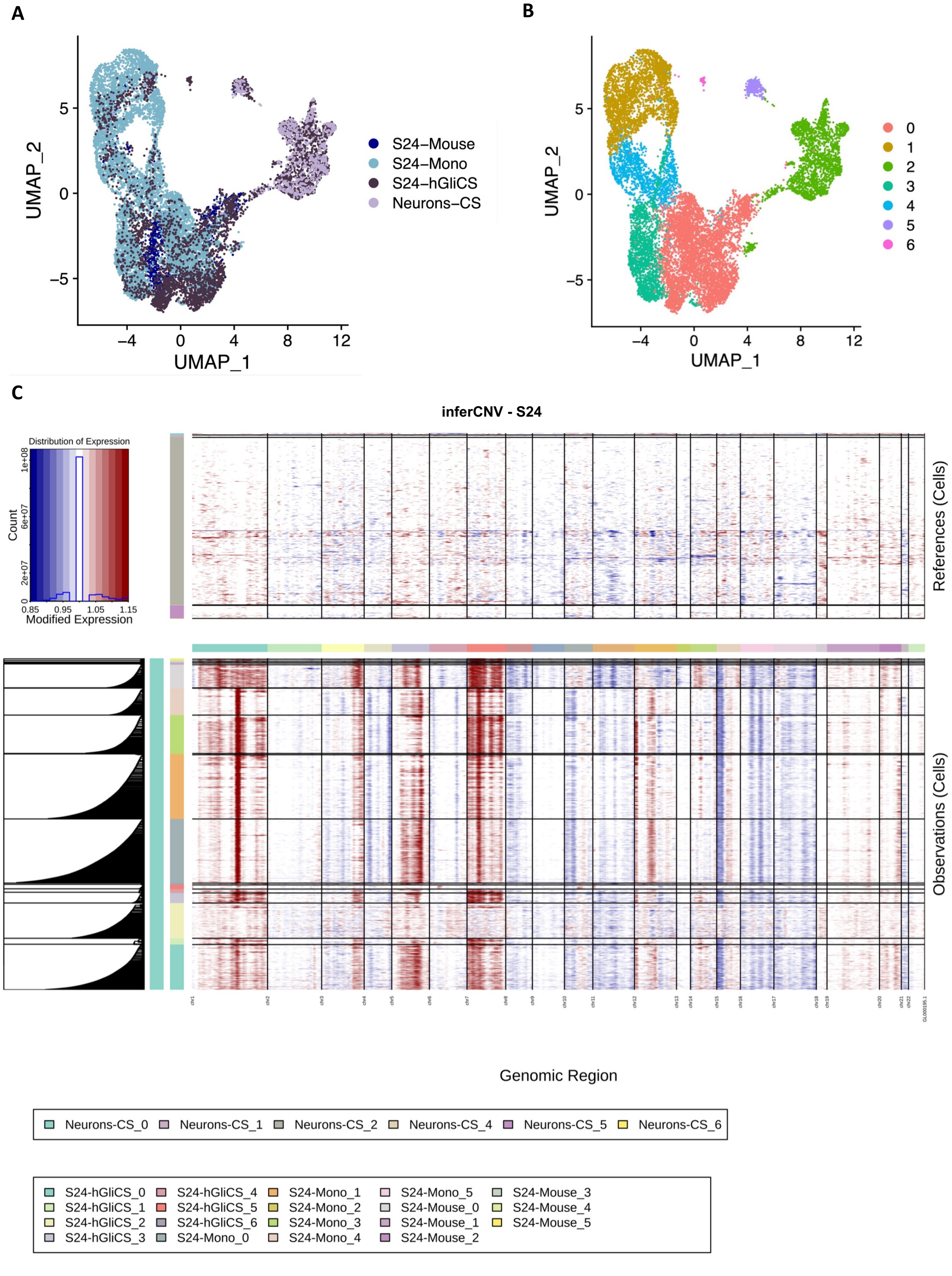

Fig. S2: Classification of malignant and non-malignant cell states for the S24 dataset.

(A) UMAP projection of GBM and neuronal cells from the S24 experiment colored by experimental condition. **(B)** UMAP projection of the S24 experiment colored by cluster (0-6) based on the Seurat clustering algorithm. **(C)** Heatmap visualization of inferCNV results for the S24 dataset confirming clustering of cells in the UMAP based on malignant/non-malignant cell state. Cells from the neuronal cortical spheroid culture (Neurons-CS) were used as the reference dataset. The top heatmap panel confirms the absence of major copy number variations in the neuronal spheroid culture. The two S24 coculture conditions (S24-hGliCS and S24-Mouse) were used as the query dataset. GBM cells in the S24 coculture conditions were identified by inferCNV based on strong chromosomal copy number variations reflected by red and blue colors in the bottom heatmap panel. Cluster labels in inferCNV contain information on condition (Figure S2A) and Seurat clusters (Figure S2B).

Supplementary Figure 3

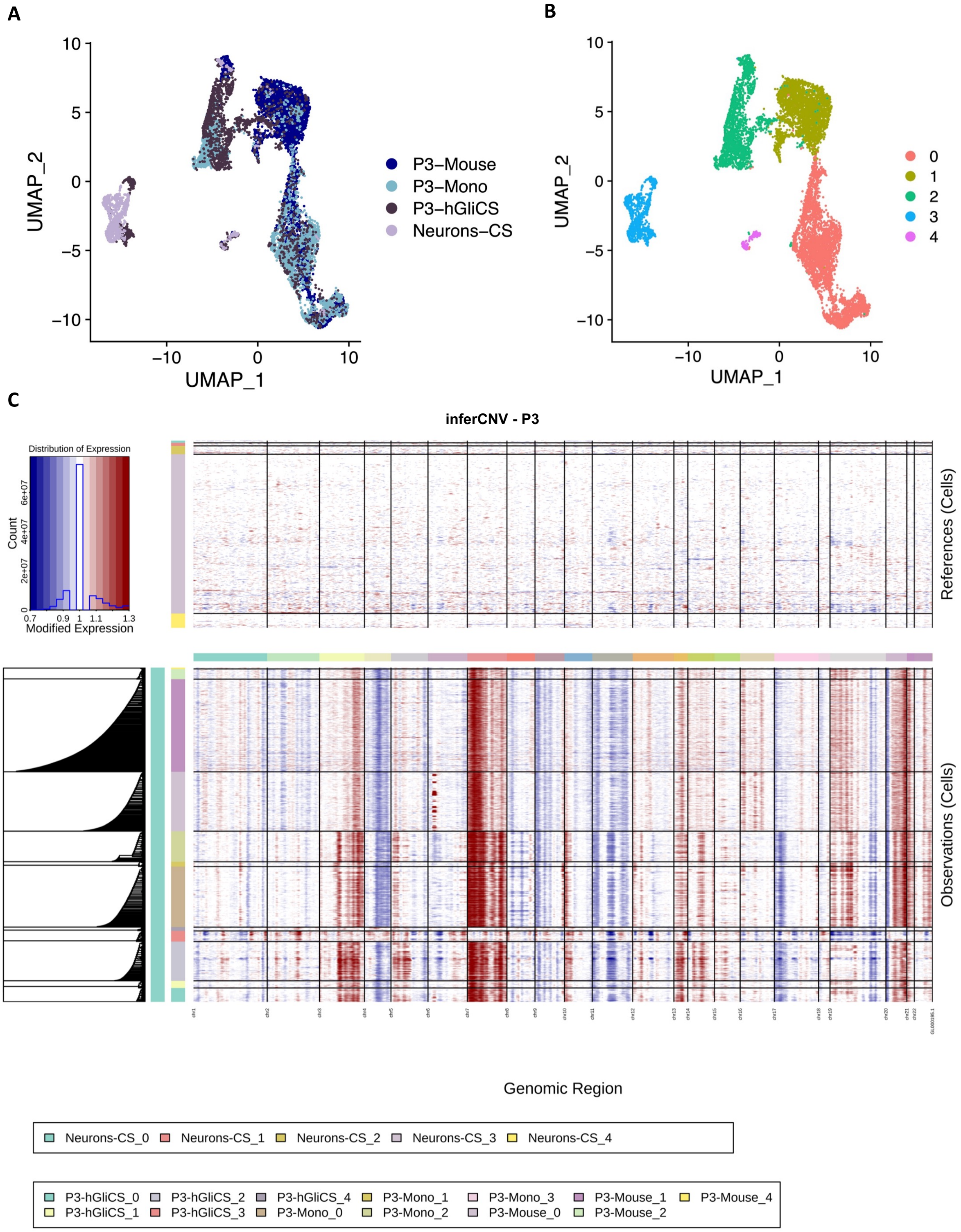

Fig. S3: Classification of malignant and non-malignant cell states for the P3 dataset.

(A) UMAP projection of GBM and neuronal cells from the P3 experiment colored by experimental condition. **(B)** UMAP projection of the P3 experiment colored by cluster (0-4) based on the Seurat clustering algorithm. **(C)** Heatmap visualization of inferCNV results for the P3 dataset. Cells from the neuronal cortical spheroid culture (Neurons-CS) were used as the reference dataset. The top heatmap panel confirms the absence of major copy number variations in the neuronal spheroid culture. The two P3 coculture conditions (P3-hGliCS and P3-Mouse) were used as the query dataset. GBM cells in the P3 coculture conditions were identified by inferCNV based on strong chromosomal copy number variations reflected by red and blue colors in the bottom heatmap panel. Cluster labels in inferCNV contain information on condition (Figure S3A) and Seurat clusters (Figure S3B).

Supplementary Figure 4

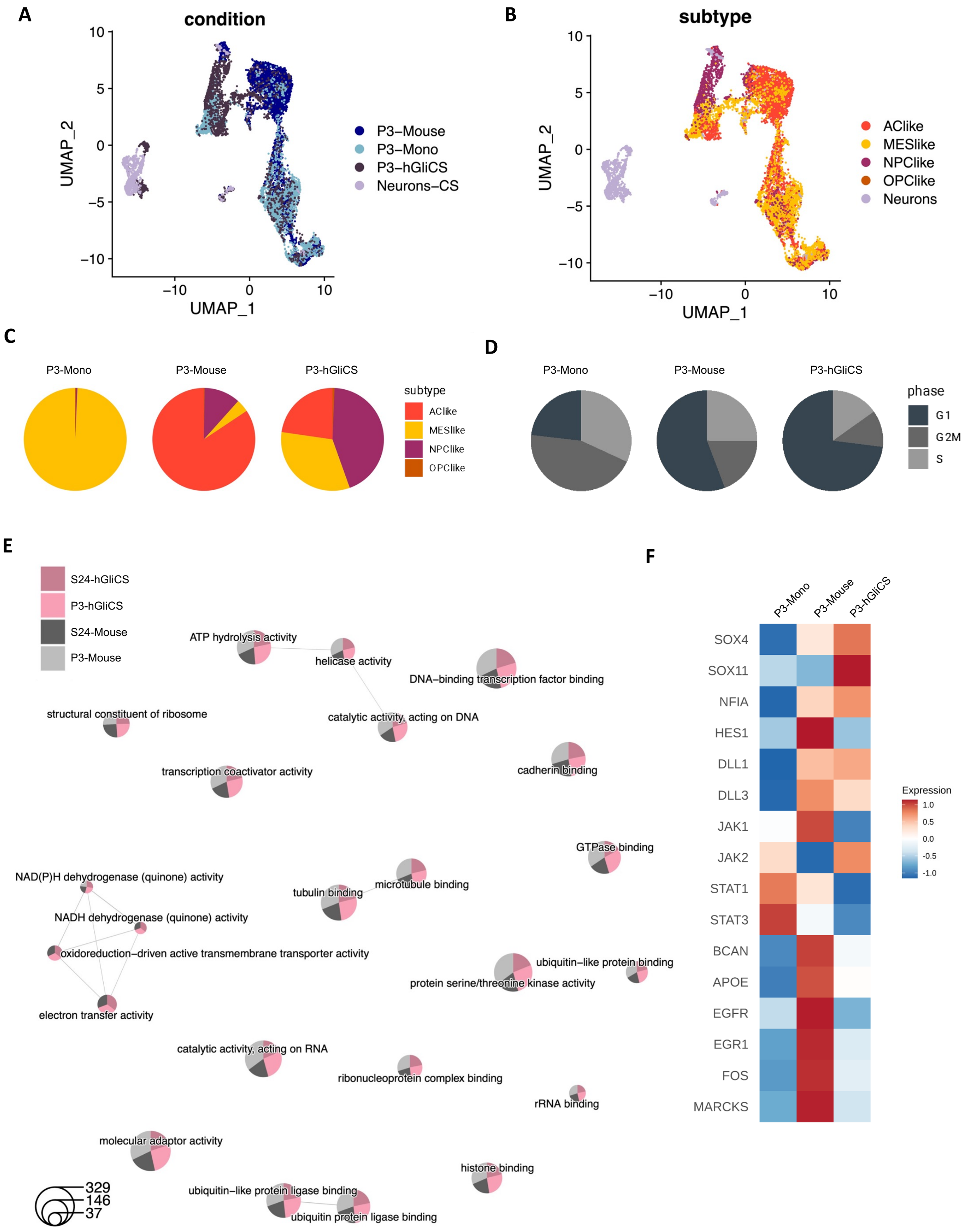

Fig. S4: Single cell transcriptomic analyses of environment-dependent models reveal high congruency between hGliCS and xenograft. Results for GBM line P3.

(A) UMAP projection of P3 GBM cell fraction across all models (P3-Mouse, P3-Mono, P3-hGliCS) and the neuronal fraction from cortical spheroids (Neurons-CS) displaying significant overlap of the three models and at the same time a clear separation of neurons from GBM cells. **(B)** UMAP projection of P3 GBM cell fraction across all models according to GBM subtypes based on Neftel gene sets (6) with the additional neuronal cluster from cortical spheroids (Neurons). **(C)** Pie charts depicting percentage of GBM subtype based on Neftel gene sets (6) only in P3 GBM cell fraction by model type. P3-Mono primarily consisted of MES-like cells, P3-Mouse predominantly of AC-like cells. P3-hGliCS displayed increased cell state diversity compared to P3-Mono. **(D)** Pie charts reflecting the distribution of cell cycle state only in P3 GBM cell fraction by model type. P3-Mono displayed a higher fraction of cycling cells compared to both, P3-hGliCS and P3-Mouse. **(E)** Combined GO analysis for both GBM lines (S24+P3) in both models (hGliCS + Mouse). Upregulated genes compared to 2D-monocultured GBM cells were analyzed in a differential gene expression analysis. The pie plot visualization indicates GO term overlap across the four comparisons and thereby suggests a high congruency between the respective hGliCS + Mouse conditions for each GBM line. **(F)** Heatmap of scaled and centered expression values for genes selected based on KEGG analysis or literature. Expression levels are provided for the P3 GBM cell fraction, in hGliCS, Mouse, and P3 monoculture conditions.

Supplementary Figure 5

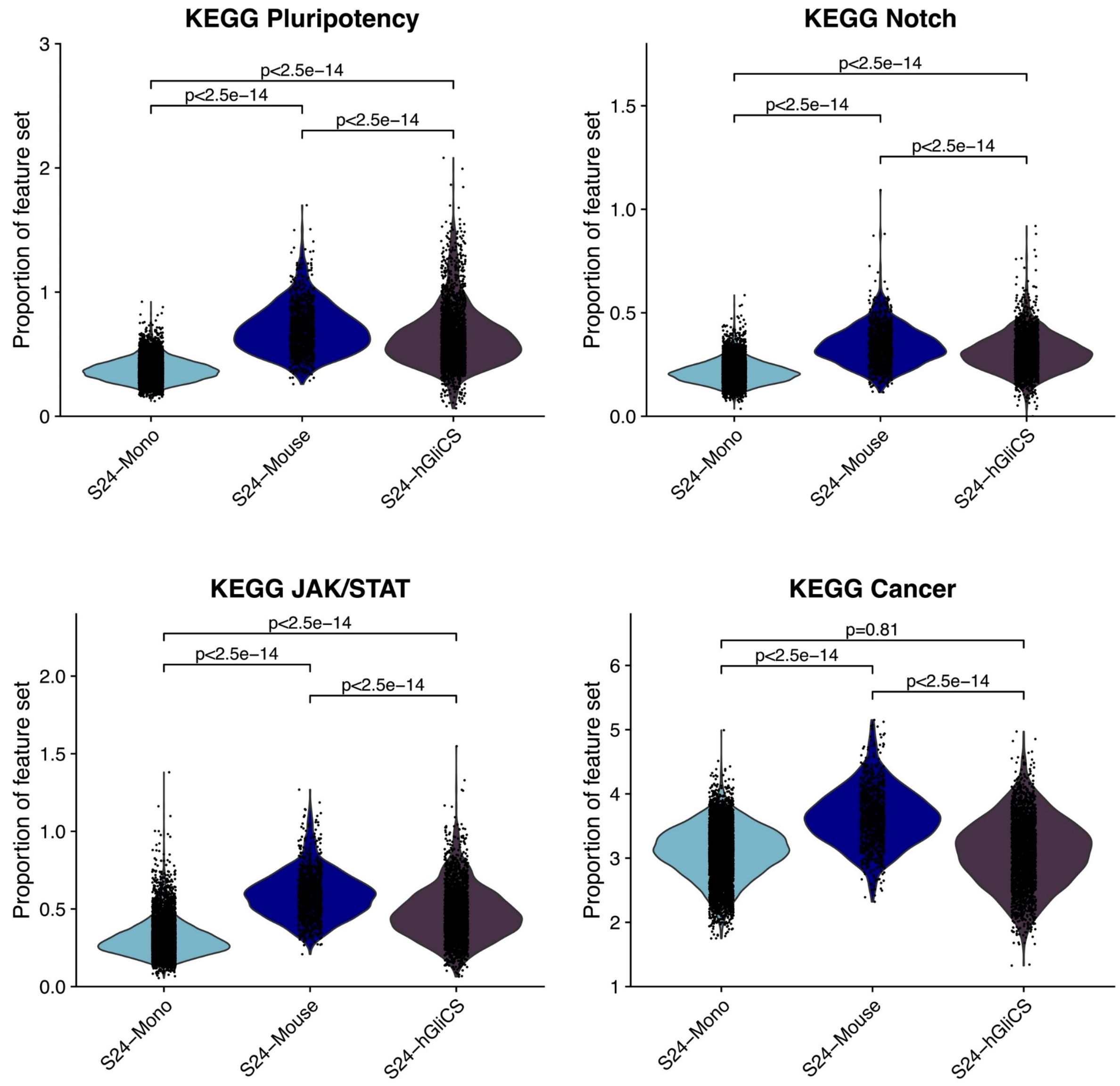

Fig. S5: Gene expression levels of complete KEGG pathway terms from Fig. 3H across all models.

Violin plots of KEGG pathways Pluripotency, Notch, JAK/STAT, and Cancer displaying the proportion of gene expression levels for the respective pathway genes in the S24 GBM fraction across all models (S24-Mono, S24-Mouse, and S24-hGliCS). The mouse xenograft and hGliCS show greater similarity in the expression of KEGG pathway genes compared to the monoculture. One-way ANOVA for multiple groups with Tukey HSD post-hoc test was performed. Tukey-adjusted p-values are indicated for each plot.

Supplementary Figure 6

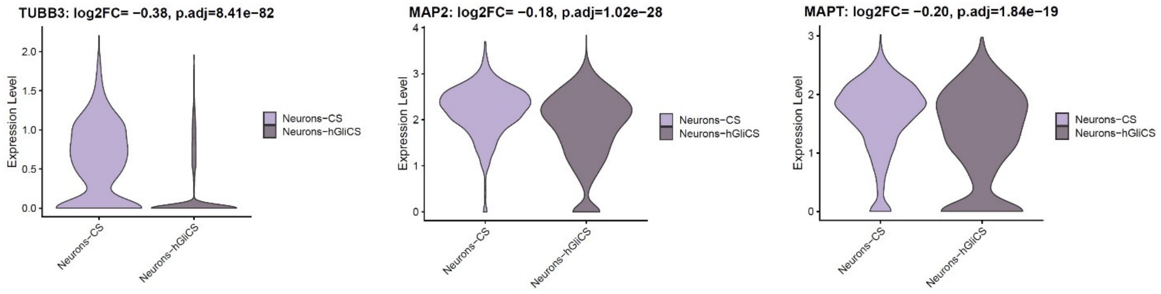

Fig. S6: Gene expression levels of selected DEGs of neurons in cortical spheroids or in hGliCS.

Violin plots of TUBB3, MAP2, and MAPT indicating expression levels in neurons of cortical spheroids (Neurons-CS) or in neurons of the hGliCS model (Neurons-hGliCS). Log2FC from Mann-Whitney-U test and p.adj-value after Bonferroni correction are provided for each gene.

Supplementary Figure 7

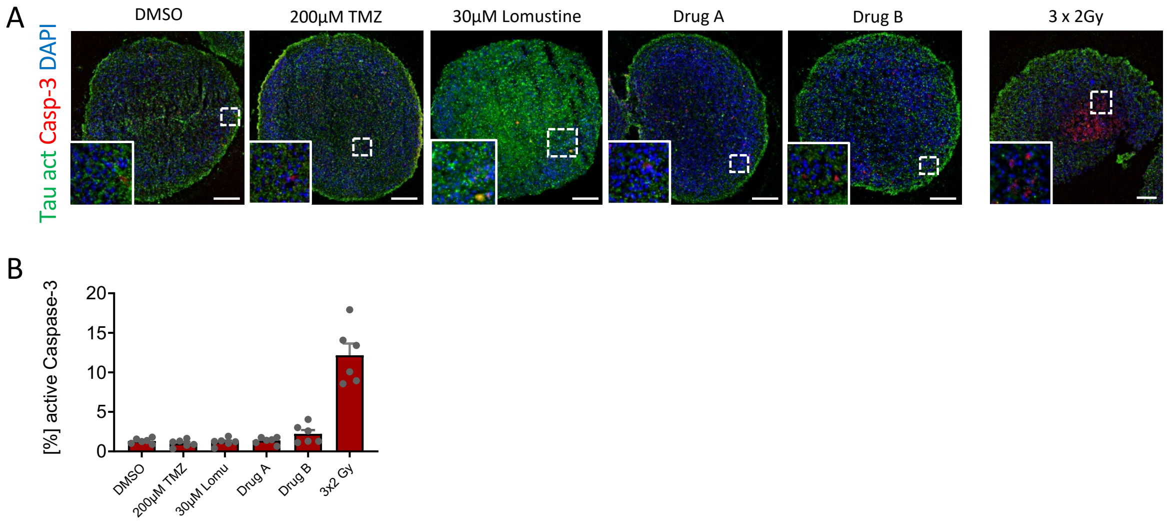

Fig. S7: Effect of cytostatic drugs on cortical spheroids.

(A) Representative immunofluorescences of cortical spheroids treated for 14 days with cytostatic drugs Temozolomide (TMZ), Lomustine, and anti-TM-drugs A + B. Stained against neuronal marker Tau (green) and apoptotic marker protein active Caspase-3 (red). In the lower left a zoom section of the indicated rectangle is shown. As positive control for the staining of active Caspase-3 an over-irradiated cortical spheroid was used (3 x 2 Gy). **(B)** Quantification of active Caspase-3 (Graph shows means±SEM from n=2 experiments, with N=3 spheroids per experiment).
